# Risk stratification using SpO_2_/FiO_2_ and PEEP at initial ARDS diagnosis and after 24 h in patients with moderate or severe ARDS

**DOI:** 10.1186/s13613-017-0327-9

**Published:** 2017-10-25

**Authors:** Luigi Pisani, Jan-Paul Roozeman, Fabienne D. Simonis, Antonio Giangregorio, Sophia M. van der Hoeven, Laura R. Schouten, Janneke Horn, Ary Serpa Neto, Emir Festic, Arjen M. Dondorp, Salvatore Grasso, Lieuwe D. Bos, Marcus J. Schultz, for the MARS consortium, Roosmarijn T. M. van Hooijdonk, Mischa A. Huson, Laura R. A. Schouten, Marleen Straat, Fabienne D. Simonis, Lieuwe D. Bos, Lonneke A. van Vught, Maryse A. Wiewel, Esther Witteveen, Gerie J. Glas, Luuk Wieske, Jos F. Frencken, Marc Bonten, Peter M. C. Klein Klouwenberg, David Ong, Brendon P. Scicluna, Arjan J. Hoogendijk, H. Belkasim-Bohoudi, Tom van der Poll

**Affiliations:** 1 Department of Intensive Care, Academic Medical Center, Meibergdreef 9, 1105 AZ Amsterdam, The Netherlands; 2 Laboratory of Experimental Intensive Care and Anesthesiology (LEICA), Academic Medical Center, Amsterdam, The Netherlands; 3 Anesthesia and Intensive Care Unit, Department of Emergency and Organ Transplantation, University of Bari Aldo Moro, Bari, Italy; 4 Department of Pediatrics, Academic Medical Center, Amsterdam, The Netherlands; 5 Department of Pulmonology, Academic Medical Center, Amsterdam, The Netherlands; 6 Department of Critical Care Medicine, Hospital Israelita Albert Einstein, São Paulo, Brazil; 7 Pulmonary and Critical Care Medicine, Mayo Clinic, Jacksonville, FL USA; 8 Mahidol–Oxford Research Unit (MORU), Faculty of Tropical Medicine, Mahidol University, Bangkok, Thailand

**Keywords:** Acute respiratory distress syndrome (ARDS), Pulse oximetry, Blood gas analysis, Positive end-expiratory pressure (PEEP), Classification, Risk stratification, Outcome, Mortality

## Abstract

**Background:**

We assessed the potential of risk stratification of ARDS patients using SpO_2_/FiO_2_ and positive end-expiratory pressure (PEEP) at ARDS onset and after 24 h.

**Methods:**

We used data from a prospective observational study in patients admitted to a mixed medical–surgical intensive care unit of a university hospital in the Netherlands. Risk stratification was by cutoffs for SpO_2_/FiO_2_ and PEEP. The primary outcome was in-hospital mortality. Patients with moderate or severe ARDS with a length of stay of > 24 h were included in this study. Patients were assigned to four predefined risk groups: group I (SpO_2_/FiO_2_ ≥ 190 and PEEP < 10 cm H_2_O), group II (SpO_2_/FiO_2_ ≥ 190 and PEEP ≥ 10 cm), group III (SpO_2_/FiO_2_ < 190 and PEEP < 10 cm H_2_O) and group IV (SpO_2_/FiO_2_ < 190 and PEEP ≥ 10 cm H_2_O).

**Results:**

The analysis included 456 patients. SpO_2_/FiO_2_ and PaO_2_/FiO_2_ had a strong relationship (*P* < 0.001, *R*_2_ = 0.676) that could be described in a linear regression equation (SpO_2_/FiO_2_ = 42.6 + 1.0 * PaO_2_/FiO_2_). Risk stratification at initial ARDS diagnosis resulted in groups that had no differences in in-hospital mortality. Risk stratification at 24 h resulted in groups with increasing mortality rates. The association between group assignment at 24 h and outcome was confounded by several factors, including APACHE IV scores, arterial pH and plasma lactate levels, and vasopressor therapy.

**Conclusions:**

In this cohort of patients with moderate or severe ARDS, SpO_2_/FiO_2_ and PaO_2_/FiO_2_ have a strong linear relationship. In contrast to risk stratification at initial ARDS diagnosis, risk stratification using SpO_2_/FiO_2_ and PEEP after 24 h resulted in groups with worsening outcomes. Risk stratification using SpO_2_/FiO_2_ and PEEP could be practical, especially in resource-limited settings.

**Electronic supplementary material:**

The online version of this article (doi:10.1186/s13613-017-0327-9) contains supplementary material, which is available to authorized users.

## Introduction

In the Berlin definition for acute respiratory distress syndrome (ARDS), risk stratification was suggested by categorizing patients as having ‘mild,’ ‘moderate’ or ‘severe’ ARDS based on the ratio of the arterial oxygen tension (PaO_2_) to the fraction of inspired oxygen (FiO_2_) at initial diagnosis [1]. This approach knows several challenges. First, it requires invasive and expensive arterial blood sampling and analysis that is frequently not available, in particular in resource-limited settings. Second, the robustness of this way of classifying patients was not confirmed by external validation [2]. Most important, though, is that the level of positive end-expiratory pressure (PEEP) may affect the PaO_2_/FiO_2_ [3] and that PaO_2_/FiO_2_ and PEEP collected at later time points, for instance 24 h after the initial ARDS diagnosis, largely improve risk stratification [4, 5].

Due to the sigmoidal nature of the oxyhemoglobin dissociation curve, the pulse oximetry saturation (SpO_2_) may serve as a reliable alternative for the PaO_2_ in patients with SpO_2_ levels lower than 97% [6]. Indeed, patients with ARDS diagnosed by means of the SpO_2_/FiO_2_ have similar characteristics and outcomes as patients in whom ARDS is diagnosed using the PaO_2_/FiO_2_ [7], and persistence of a high oxygen saturation index at 24 h has been found to be associated with worse outcomes in cases of pediatric ARDS [8]. As pulse oximetry is noninvasive, inexpensive and widely available, SpO_2_/FiO_2_ could serve as an attractive alternative for PaO_2_/FiO_2_ in risk stratification of ARDS patients.

The overarching aim of the present investigation was to determine whether classification of patients using SpO_2_/FiO_2_ and PEEP at initial ARDS diagnosis and after 24 h could be used to stratify for risk of mortality. Specifically, we hypothesized that the SpO_2_/FiO_2_ could serve as an alternative for the PaO_2_/FiO_2_ in risk classification and that re-classification using SpO_2_/FiO_2_ and PEEP after 24 h improves risk stratification in a cohort of consecutive adult patients with moderate or severe ARDS in an intensive care unit (ICU) in the Netherlands.

## Methods

### Study design

We used data from a prospective observational conveniently sized cohort of well-defined critically ill patients admitted to the mixed surgical–medical intensive care unit of one university hospital in the Netherlands [9]. These data were matched with nurse-validated oxygenation data at baseline and after 24 h that was retrospectively collected from the patient data management system (Metavision^®^, iMDSoft, Tel Aviv, Israel). The Institutional Review Board of the Academic Medical Center approved the parent study protocol and use of an ‘opt-out’ consent method (IRB no. 10-056C).

### Inclusion and exclusion criteria

A team of trained ICU researchers scored patients for the absence or presence of ARDS according to the criteria stated by the American–European Consensus Conference on ARDS [10]. Although this study started in 2011 (i.e., before publication of the Berlin definition for ARDS [1]), all patients diagnosed with ARDS fulfilled the criteria of the latest definition for ARDS. Patients with mild ARDS were excluded as were patients discharged or transferred to another ICU before 24 h after the initial ARDS diagnosis.

### Outcomes

The primary outcome was all-cause in-hospital mortality. Secondary outcomes included ICU mortality, 30- and 90-day, and 1-year mortality, and the number of ventilator-free days and alive at day 28 (VFD-28), defined as the number of days from days 1 to 28 that the patient is alive and breathing without invasive assistance of the mechanical ventilator for at least 24 consecutive hours.

### Data collection

At initial ARDS diagnosis and after 24 h, pulse oximetry results were retrospectively collected, and the corresponding SpO_2_/FiO_2_ was calculated. For this, we first collected ten successive nurse-validated SpO_2_ and FiO_2_ values over 10 min directly preceding, and at the time, an arterial blood sample was drawn for blood gas analysis. The median of these ten SpO_2_ and FiO_2_ values was used to calculate the SpO_2_/FiO_2_ to alleviate potential artifactual SpO_2_ values. In all patients, pulse oximetry was measured by conventional two-wavelength finger pulse oximeters. The local protocol dictated a change in finger position every 3–4 h to avoid decubitus lesions and to use alternative probes, such as nose and forehead probes, only in patients in whom no adequate signal could be obtained with a conventional pulse oximeter on a finger. In addition to this, PaO_2_ and PaCO_2_ levels, pH levels and body temperature were collected, and it was determined whether or not patients received treatment with vasopressors at both time points.

Baseline data collected from the original database included demographic and ventilator data, comorbidities, admission type, patient category and cause of ARDS. The Acute Physiology and Chronic Health Evaluation (APACHE) IV score [11], the Lung Injury Prediction Score [12] and Charlson comorbidity score [13] were calculated.

### Analysis plan

The correlation between SpO_2_/FiO_2_ and PaO_2_/FiO_2_ was studied only using SpO_2_/FiO_2_ and PaO_2_/FiO_2_ at initial ARDS diagnosis. SpO_2_ values of > 97% were excluded from the correlation analysis as the oxyhemoglobin dissociation curve flattens above this level and large changes in the PaO_2_ may result in little or no change in the SpO_2_, in line with previous investigations that analyzed this relationship [6, 14, 15]. A nonlinear equation (the ‘Ellis formula’) was also used to estimate PaO_2_/FiO_2_ from SpO_2_ and FiO_2_ values [16, 17].

Next, we classified all patients at initial ARDS diagnosis and re-classified patients after 24 h, into four risk groups based on predefined SpO_2_/FiO_2_ and PEEP cutoffs. As two recent studies of risk stratification of ARDS patients used a cutoff for the PaO_2_/FiO_2_ of 150 mmHg [4, 5], which corresponds to a SpO_2_/FiO_2_ of 190 [6], we used this value as a cutoff for SpO_2_/FiO_2_ in the present analysis. We used the same cutoff for PEEP as in two previous studies [4, 5]. Accordingly, we created four groups: SpO_2_/FiO_2_ ≥ 190 and PEEP < 10 cm H_2_O (group I); SpO_2_/FiO_2_ ≥ 190 and PEEP ≥ 10 cm (group II); SpO_2_/FiO_2_ < 190 and PEEP < 10 cm H_2_O (group III); and SpO_2_/FiO_2_ < 190 and PEEP ≥ 10 cm H_2_O (group IV). The comparison among groups included all pairwise comparisons across the four risk groups.

### Statistical analysis

Data were expressed as mean ± standard deviation (SD), median with interquartile range (IQR) or number with percentage, where appropriate.

A two-way scatterplot and Pearson correlation analysis were used to characterize the relationship between SpO_2_/FiO_2_ (linear and nonlinear estimations) and PaO_2_/FiO_2_. Linear regression allowed quantification of the best regression line and derive a predictive equation for the relationship. Hence, based on the derived regression equation, we obtained the SpO_2_/FiO_2_ values that correspond to PaO_2_/FiO_2_ ratio values of 100, 150 and 200 mmHg. PaCO_2_, arterial pH, body temperature, PEEP and use of vasopressors were tested as interaction terms in the model to evaluate moderation of the association between SpO_2_/FiO_2_ and PaO_2_/FiO_2_. The area under the receiver operating characteristic (ROC) curve evaluated the applicability of SpO_2_/FiO_2_ in discriminating moderate from severe ARDS.

Differences between risk groups were tested with the Pearson Chi-square or Fisher exact test for categorical variables and with one-way ANOVA or Kruskal–Wallis test for continuous variables. In-hospital mortality and other mortality endpoints were calculated for each of the four groups, and a *P* value for trend was calculated from a chi-squared test for trend in proportions (i.e., the Cochrane–Armitage test), testing the null hypothesis that the proportions in several groups are the same. A pairwise comparison at ARDS diagnosis and after 24 h was performed using a contrast matrix predictor approach in which odd ratios (ORs) and 95% confidence intervals are generated for each mutual comparison between groups.

### Post hoc analyses

Several post hoc analyses were performed. First, we evaluated whether transforming the data to fractions (1/PaO_2_/FiO_2_ and 1/SpO_2_/FiO_2_) improved the fit between SpO_2_/FiO_2_ and PaO_2_/FiO_2_, as previously reported [18].

Second, risk classification using the Berlin definition threshold for severe ARDS (i.e., 100 mm Hg) [6] was performed.

Third, in the last post hoc analysis we analyzed whether the association between risk stratification and in-hospital mortality was confounded by factors such as disease severity, and other readily available parameters in the database, such as arterial pH and plasma lactate levels, blood pressure levels and vasopressor therapy. For this analysis, a multivariable logistic regression model was built to assess the confounding effect of these variables on the association between risk groups and the primary outcome.

All analyses were performed in R via the R-studio interface (R version 3.0, www.r-project.org). A *P* value below 0.05 was considered significant.

## Results

### Patients

**Fig. 1:**
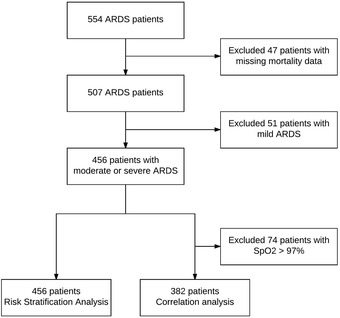
Study flowchart. SpO_2_, pulse oximetry oxygen saturation; ARDS, acute respiratory distress syndrome

Of 554 patients with ARDS, 456 were classified as having moderate or severe ARDS according to the Berlin definition and had complete datasets. Of them, 382 could be used for the correlation analyses, and all patients for the classification and re-classification analyses (Fig. 1). Pneumonia, sepsis, major surgery and trauma were the most common causes of ARDS (Table 1). At baseline, there were no differences between survivors and non-survivors with regard to oxygenation parameters, tidal volume size, the Lung Injury Prediction Score and the etiology of ARDS. Also, there were no differences with regard to the comorbidity score. Overall all-cause in-hospital mortality rate was 39.7%. Non-survivors were older, had a lower arterial pH levels at ARDS diagnosis, were ventilated at higher respiratory rates and had higher disease severity scores. Berlin definition class distribution was not different between survivors and non-survivors.

**Table 1:** Baseline characteristics of 456 survivors and non-survivors with moderate and severe ARDS

Variables	All patients	Survivors *N* = 275	Non-survivors *N* = 181	*P* value
Age, years	62 (51–72)	60 (48–70)	63 (55–72)	0.008
Female		113 (41.1)	59 (32.6)	0.075
Weight, kg	80 (70–90)	80 (70–90)	80 (68–87)	0.347
APACHE IV	68 (51–89)	72 (58–95)	90 (74–113)	< 0.001
Charlson comorbidity index 2	1 (0–3)	1 (0–2)	1 (0–3)	0.103
Lung Injury Prediction Score	8.5 (7–10)	8 (6.5–10)	9 (7–10)	0.074
Tidal volume, ml/kg	6.3 (5.3–7.6)	6.3 (5.3–7.8)	6.3 (5.4–7.3)	0.412
Respiratory rate	23 (17–29)	20 (16–27)	25 (19–30)	0.001
PEEP, cmH_2_O	10 (8–12)	10 (8–12)	10 (8–13)	0.436
FiO_2_, %	60 (50–75)	60 (50–70)	60 (50–80)	0.462
Arterial pH	7.34 (7.26–7.41)	7.35 (7.28–7.41)	7.31 (7.22–7.39)	0.001
PaCO2, kPa	5.8 (5.1–6.9)	5.7 (5–6.9)	6 (5.2–7.3)	0.072
PaO_2_/FiO_2_	120 (89–149)	123 (91–149)	115 (89–149)	0.353
SpO_2_/FiO_2_	160 (126–190)	163 (127–191)	157 (124–190)	0.344
Vasopressor use in first 24 h	293 (64.3)	164 (59.6)	129 (71.3)	0.015
ARDS class				
Moderate ARDS	300 (65.8)	181 (65.8)	119 (65.7)	1
Severe ARDS	156 (34.2)	94 (34.2)	62 (34.3)	1
Cause of ARDS				
Pulmonary origin	325 (71.3)	200 (72.7)	125 (69.1)	0.732
Non-pulmonary origin	185 (40.6)	104 (37.8)	81 (44.8)	0.382

Categorical variables: number (percentage); continuous variables: median (25–75 percentile)

*PEEP* positive end-expiratory pressure, *FiO2* fraction of inspired oxygen, *PaCO*_*2*_ arterial carbon dioxide tension, *PaO*_*2*_ arterial oxygen tension, *SpO*_*2*_ pulse oximetry saturation, *ARDS* acute respiratory distress syndrome, *APACHE IV* acute physiology and chronic evaluation IV

### Relationship between SpO_2_/FiO_2_ and PaO_2_/FiO_2_

**Fig. 2:**
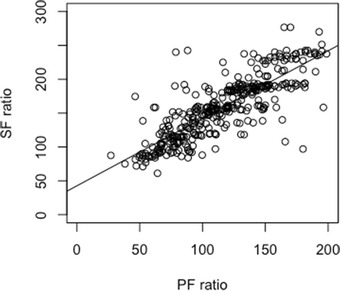
Scatterplot of SPO_2_/FiO_2_ versus PaO_2_/FiO_2_ at initial ARDS diagnosis. The line represents the best-fit linear relationship: SpO_2_/FiO_2_ = 42.6 + 1.0 * PaO_2_/FiO_2_ [*P* < 0.001, *R* ^2^ = 0.676] at initial ARDS diagnosis

The correlation between SpO_2_/FiO_2_ and PaO_2_/FiO_2_ was strong (*P* < 0.001, *R* ^2^ = 0.676; Fig. 2) and could be described in a linear regression equation:

$${\text{SpO}}_{2} / {\text{FiO}}_{2} = 42.6 + 1.00 * {\text{PaO}}_{2} / {\text{FiO}}_{2}$$

This relationship was neither moderated by arterial PaCO_2_ and PEEP, nor by body temperature and vasopressor therapy. There was a moderation effect by arterial pH and FiO_2_, as the steepness of the slope between PaO_2_/FiO_2_ and SpO_2_/FiO_2_ was inclined by arterial pH (1.4, *P* < 0.001) and declined by FiO_2_ (− 0.01, *P* < 0.001).

Based on the found equation, a PaO_2_/FiO_2_ of 200 mm Hg corresponded to a SpO_2_/FiO_2_ of 243 [95% CI 220–265], a PaO_2_/FiO_2_ of 150 mm Hg to a SpO_2_/FiO_2_ of 193 [95% CI 174–211] and a PaO_2_/FiO_2_ of 100 mm Hg to a SpO_2_/FiO_2_ of 143 [95% CI 127–158].

**Fig. 3:**
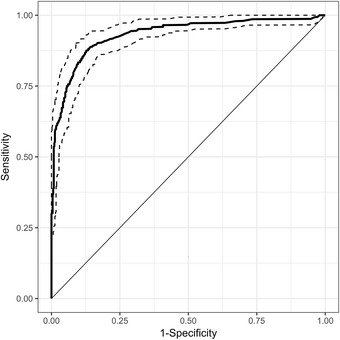
ROC curve for SpO_2_/FiO_2_ versus PaO_2_/FiO_2_ < 100. Dotted lines represent 95% confident intervals, AUC = 0.928. AUC, area under the curve

The SpO_2_/FiO_2_ had an excellent ability to discriminate moderate from severe ARDS, with a ROC area under the curve of 0.928 (Fig. 3). The nonlinear formula (Ellis formula) to estimate the PaO_2_/FiO_2_ from the SpO_2_ and FiO_2_ was not superior to the linear model when SpO_2_ ≤ 97% (*N* = 382, *R* ^2^ = 0.656).

The use of fractions (1/SpO_2_/FiO_2_ and 1/PaO_2_/FiO_2_) did not result in a stronger linear relationship (*R* ^2^ = 0.629). The regression equation, though, was very similar to one previously reported in pediatric patients (1/SpO_2_/FiO_2_ = 0.0024 + 0.46/PaO_2_/FiO_2_) [18].

### Risk stratification

The distribution and outcome data for the four risk groups are presented in Table 2. There was no trend for increasing in-hospital mortality rate among the four risk groups at baseline (*P* value for trend = 0.90). A pairwise comparison also showed no significant differences in hospital mortality between groups at ARDS diagnosis. In contrast, the same categorization after 24 h resulted in risk groups with increasing rates of in-hospital mortality (*P* value for trend < 0.001). A pairwise comparison showed that the differences in in-hospital mortality between group IV and group II (OR 2.40 [1.15–4.82]; *P* value = 0.012), and between group IV and group I were significant (OR 2.47 [1.26–4.85]; *P* value = 0.003) (Table 3).

**Table 2:** Distribution and outcomes of each subset of patients with ARDS at initial diagnosis and after 24 h

Outcome	Group I	Group II	Group III	Group IV	*P* value
	SpO_2_/FiO_2_ ≥ 190 and PEEP < 10	SpO_2_/FiO_2_ ≥ 190 and PEEP ≥ 10	SpO_2_/FiO_2_ < 190 and PEEP < 10	SpO_2_/FiO_2_ < 190 and PEEP ≥ 10	
At onset of ARDS (N)	87	43	100	226	
ICU mortality (%)	19.5	23.3	18.0	30.1	0.042
In-hospital mortality (%)	44.8	30.2	36.0	41.2	0.897
30-day mortality (%)	27.6	27.9	24.0	33.2	0.276
90-day mortality (%)	47.1	34.9	41.0	42.0	0.636
1-year mortality (%)	60.9	41.9	47.0	49.6	0.182
VFD (days) (IQR)	19 (7–24)	22 (6.5–25)	20.5 (6–25)	18 (0–23)	0.070
After 24 h (N)	213	145	17	81	
ICU mortality (%)	15.5	24.1	35.3	48.2	< 0.001
In-hospital mortality (%)	34.7	35.9	52.9	56.8	< 0.001
30-day mortality (%)	23.9	26.9	47.1	45.7	< 0.001
90-day mortality (%)	38.0	38.6	52.9	56.8	0.003
1-year mortality (%)	47.4	45.5	58.8	65.4	0.006
VFD 28 (days) (IQR)	23 (15–26)	18 (0–23)	6 (0–17)	0 (0–17)	< 0.001

*VFD-28* ventilator-free days and alive at day 28, *IQR* interquartile range, *CI* confidence interval

**Table 3:** Intergroup comparisons at ARDS diagnosis and after 24 h for in-hospital mortality

Comparison	At ARDS diagnosis		After 24 h	
	OR (95% CI)	*P* value	OR (95% CI)	*P* value
Group II versus I	0.53 (0.19–1.46)	0.376	1.05 (0.59–1.86)	0.996
Group III versus I	0.69 (0.32–1.49)	0.600	2.11 (0.59–7.62)	0.433
Group IV versus I	0.86 (0.45–1.65)	0.933	2.47 (1.26–4.85)	0.003
Group III versus II	1.30 (0.48–3.53)	0.907	2.01 (0.55–7.43)	0.509
Group IV versus II	1.61 (0.65–4.03)	0.531	2.40 (1.15–4.82)	0.012
Group IV versus III	1.24 (0.66–2.34)	0.811	1.17 (0.30–4.53)	0.991

*ARDS* acute respiratory distress syndrome, *OR* odd ratio, *CI* confidence interval

Findings were comparable for the secondary outcomes. While classification using cutoffs for SpO_2_/FiO_2_ and PEEP at initial ARDS diagnosis were not related to any of these outcomes, risk stratification at 24 h resulted in significant positive trends across groups, and significantly worse secondary outcomes in group IV compared to group I and to group II (Additional file 1: Table E1).

Distribution of patients in each subset noticeably changed at 24 h. Less than a quarter of patients maintained to have SpO_2_/FiO_2_ < 190; their in-hospital mortality was much higher than that of patients with a SpO_2_/FiO_2_ ≥ 190 (56.1 vs. 35.2%, *P* = 0.001). Half of the patients were ventilated at PEEP < 10 cm H_2_O; their in-hospital mortality was similar to that of patients who were kept at PEEP ≥ 10 cm H_2_O (36.1 vs. 43.4 *P* = 0.107). When considering the characteristics of the four risk groups at 24 h, we found statistical differences in the APACHE IV score, arterial pH and FiO_2_ at 24 h, maximum plasma lactate levels and vasopressor use, which could explain the significant differences in outcome (Table 3).

### Post hoc analyses

The post hoc analysis with the adapted SpO_2_/FiO_2_ cutoff of 150, in line with the Berlin definition cutoff of PaO_2_/FiO_2_ of 100 mm Hg, showed similar results to the primary analysis, albeit group sizes changed. No trends in risk were observed other than for ICU mortality when using this cutoff at onset of ARDS, while a significant trend across the four risk groups was noted using this cutoff after 24 h (Additional file 1: Table E2). The intergroup comparisons also mirrored the primary analysis, with no significant contrasts at ARDS diagnosis, but clear contrast after 24 h (group IV vs. group I and II) (Additional file 1: Table E3).

The multivariable model showed that APACHE IV scores, plasma lactate levels and arterial pH significantly confounded the relationship between the group stratification and in-hospital mortality (Additional file 1: Table E4). The same applied when restricting this analysis to the extreme risk groups (i.e., groups I and IV), in which even vasopressor therapy was a significant confounder (Additional file 1: Table E5).

## Discussion

To our best knowledge, this is the first study in which ARDS patients were classified using predefined cutoff values for SpO_2_/FiO_2_ and PEEP. The most relevant findings of this study are: (1) SpO_2_/FiO_2_ confirms to be a valid surrogate for PaO_2_/FiO_2_ in patients with moderate or severe ARDS; (2) re-classification after 24 h using predefined cutoffs for SpO_2_/FiO_2_ and PEEP improves risk stratification compared to the same cutoffs at baseline; and (3) the association between risk group and outcome, however, is confounded by disease severity, arterial pH and lactate levels, and vasopressor therapy. We believe that the approach of using SpO_2_/FiO_2_ and PEEP to classify patients could be useful for the implementation of an individualized approach for appropriate diagnosis and therapy in ARDS patients, in particular in settings where it is difficult, if not impossible, to perform (repeated) blood gas analyses (Table 4).

**Table 4:** Main characteristics of 456 patients with ARDS—classification 24 h after initial ARDS diagnosis based on SpO_2_/FiO_2_ cutoff of 190 and PEEP cutoff of 10 cm H_2_O

Variables	Group I (*N* = 213)	Group II (*N* = 145)	Group III (*N* = 17)	Group IV (*N* = 81)	*P* value
	SpO_2_/FiO_2_ ≥ 190 and PEEP < 10	SpO_2_/FiO_2_ ≥ 190 and PEEP ≥ 10	SpO_2_/FiO_2_ < 190 and PEEP < 10	SpO_2_/FiO_2_ < 190 and PEEP ≥ 10	
APACHE IV score	75 (60–93)	84 (65–108)	78 (65–112)	86 (68–110)	0.001
Age, years	63 (53–73)	60 (49–69)	57 (42–70)	59 (49–69)	0.018
Female	80 (37.6)	61 (42.1)	5 (29.4)	26 (32.1)	0.436
CCI 2	1 (0–3)	1 (0–2)	2 (0–3)	1 (0–2)	0.423
LIPS	8 (6.5–9.5)	9 (7.5–10.5)	7 (6.5–8)	9.5 (7.5–11.5)	< 0.001
Tidal volume, ml/kg	6.4 (5.3–7.7)	6.2 (5.2–7.5)	7.2 (6.5–8.2)	6.4 (5.6–7.4)	0.166
FiO_2_, %	40 (36–40)	40 (40–50)	60 (55–60)	60 (55–65)	< 0.001
PEEP, cm H_2_O	5 (5–8)	12 (10–13)	7 (5–8)	14 (12–15)	< 0.001
Arterial pH	7.4 (7.4–7.5)	7.4 (7.3–7.4)	7.4 (7.3–7.5)	7.3 (7.3–7.4)	< 0.001
PaCO_2_, kPa	5.3 (4.7–6.2)	5.6 (4.9–6.2)	5.6 (5–7)	5.8 (5.1–6.6)	0.018
Plasma lactate level, mmol/l	2.4 (1.4–4.1)	3.1 (1.7–6.2)	1.6 (1.4–3.9)	5.4 (2.6–10.9)	< 0.001
Respiratory rate	22 (17–27)	24 (17.2–30)	22 (18–28)	24 (16–34)	0.183
PaO_2_/FiO_2_	207 (175–252)	193 (167–226)	128 (120–149)	133 (105–153)	< 0.001
SpO_2_/FiO_2_	243 (231–265)	232 (200–245)	165 (154–170)	158 (136–176)	< 0.001
Vasopressor use in first 24 h	100 (47.0)	102 (70.3)	6 (35.3)	67 (82.72)	< 0.001
ARDS class after 24 h					
Mild ARDS	122 (57.3)	61 (42.1)	0 0	5 (6.2)	< 0.001
Moderate ARDS	91 (42.7)	84 (57.9)	15 (88.2)	59 (72.8)	< 0.001
Severe ARDS	0 0	0 0	2 (11.8)	17 (21)	< 0.001
Cause of ARDS					
Pulmonary	156 (73.2)	99 (68.3)	12 (70.6)	58 (71.6)	0.509
Non-pulmonary	67 (31.5)	78 (53.8)	5 (29.4)	40 (49.4)	0.426

Categorical variables: number (percentage); continuous variables: median (25–75 percentile)

*CCI* Charlson Comorbidity Index, *LIPS* Lung Injury Prediction Score, *APACHE* acute physiology and chronic health evaluation, *PEEP* positive end-expiratory pressure, *FiO*_*2*_ fraction of inspired oxygen, *PaCO*_*2*_ arterial carbon dioxide tension, *PaO*_*2*_ arterial oxygen tension, *SpO*_*2*_ pulse oximetry oxygen saturation, *ARDS* acute respiratory distress syndrome

Our study has several strengths. The prospective design of the collection of data, the completeness of follow-up and the fact that the ARDS diagnosis was scored by a team of trained ICU researchers prevented against bias. In addition, we could re-categorize patients from the criteria stated by the American–European Consensus Conference on ARDS [10] to the newer Berlin definition for ARDS [1], after we could select patients with moderate or severe ARDS. Finally, the number of participants was large, patients were homogeneous regarding their clinical characteristics as well as type of ARDS and the overall in-hospital mortality rate of our cohort was comparable to the pooled mortality for moderate and severe ARDS patients in the recently published LUNG SAFE study [19].

SpO_2_/FiO_2_ is an increasingly appreciated parameter in the diagnosis and management of patients with ARDS [6, 15, 17, 20, 21]. The current pediatric ARDS definition includes oximetry-based measures in preference of the PaO_2_/FiO_2_, captured in the oxygenation saturation index [22]. Unfortunately, mean airway pressures, necessary for calculation of the oxygenation saturation index, were not reliably captured in our cohort preventing us from comparing our results to those from previous studies in children [8, 18, 23]. We used a previously reported equation (SpO_2_/FiO_2_ = 64 + (0.84 * PaO_2_/FiO_2_) [6] to convert a previously used cutoff for PaO_2_/FiO_2_ (i.e., 150 mm Hg) to a cutoff for SpO_2_/FiO_2_ of 190. Using the equation that came from our own data a very similar cutoff for SpO_2_/FiO_2_ would have been chosen. Due to its increased feasibility, the SpO_2_/FiO_2_ was actually considered for inclusion during the Berlin criteria definition process [24], but was successively excluded on the possibility that it may misclassify mild into severe ARDS patients. A cutoff for PaO_2_/FiO_2_ of 150 mm Hg has frequently been shown to accurately stratify for in-hospital mortality [4, 5, 25] and was also used in two of the largest, and ultimately positive, randomized controlled trials in ARDS patients [26, 27].

The present findings on the relationship between SpO_2_/FiO_2_ and PaO_2_/FiO_2_ mirror results from previous investigations in adult [6, 15] and pediatric patients [14, 18, 23, 28], albeit that a marginally different regression equation was found. One investigation did not find a strong relationship between SpO_2_/FiO_2_ and PaO_2_/FiO_2_, but in that study the data were collected from automated anesthesia information system with a very high portion of the data excluded due to high SpO_2_ values [29]. Recently, the linear relation between SpO_2_/FiO_2_ and PaO_2_/FiO_2_ was re-challenged and both a fractional transformation [14] and a nonlinear imputation method [17, 30] were proposed to improve the model fit or better represent the sigmoidal shape of the oxyhemoglobin dissociation curve. Despite the clear physiological rationale, these approaches did not improve the relationship in the present dataset.

It has been described before that FiO_2_ at levels > 70% alters the PaO_2_/FiO_2_: an increase in FiO_2_ > 70% gradually increases the PaO_2_/FiO_2_ [31], particularly at low levels of shunt fraction [32]. The effect of FiO_2_ on SpO_2_/FiO_2_, however, could be opposite as pulse oximetry has an intrinsic upper limit much tighter than PaO_2_. An increase in FiO_2_ will gradually decrease the SpO_2_/FiO_2_. This intrinsic mathematical limitation of the SpO_2_/FiO_2_ can possibly lead to misclassification of individual ARDS cases. Of note, FiO_2_ > 70% was used in 25% of patients at ARDS diagnosis, and only in 3% after 24 h.

The level of PEEP is known to be particularly relevant as the evolution and prognosis of ARDS are related to changes in PaO_2_/FiO_2_ in response to changes in PEEP greater than or equal to 10 cm H_2_O [3, 33, 34]. While PEEP does not affect the oxyhemoglobin curve and may not significantly alter the relationship between SpO_2_ and PaO_2_ [15], it may impact the SpO_2_/FiO_2_ ratio by improving ventilation–perfusion matching and thus was considered in our analysis. Of note, the approach of using PaO_2_/FiO_2_, or SpO_2_/FiO_2_, and PEEP is not new. Indeed, risk classification in the Berlin definition uses PaO_2_/FiO_2_ and PEEP levels [1], though only at onset of ARDS and not after 24 h of standard care. The empathic aim of the present investigation was to see whether two easy to collect and almost always-available ventilator parameters, even in resource-limited settings, i.e., SpO_2_/FiO_2_ and PEEP, would alter risk groups.

Despite several classification and prediction systems for ARDS patients [1, 3, 25, 33, 35–37], we still lack a proper classification system for clinical management and research purposes that can serve as a practical model for setting individual therapeutic targets. Several investigations have shown that the PaO_2_/FiO_2_ measured at onset of ARDS cannot be used for risk stratification in patients with moderate or severe ARDS [35, 38–41]. Recently, it was shown that standardization of ventilator settings at the moment of collecting PaO_2_/FiO_2_ data [33] as well as re-categorization based on both PaO_2_/FiO_2_ and PEEP level cutoffs, measured 24 h after the initial ARDS diagnosis, largely improves risk stratification for hospital mortality [4, 5]. The findings of the present study are in agreement with and extend these findings showing for the first time that pulse oximetry after 24 h perform better than at the initial ARDS diagnosis in short- and long-term outcome stratification in adult patients with moderate or severe ARDS. Similar findings derive from the pediatric population, where oximetry derived indexes after 24 h reliably stratified outcome while initial values were not helpful in prognostication [8].

In the studied cohort of patients with moderate or severe ARDS, improvement or worsening of the SpO_2_/FiO_2_ in the first 24 h was strongly associated with outcome. Group I represents the less complicated patients with ARDS and a great portion of our patients fit in this group after 24 h (46.7 vs. 19.1% at baseline), underlining the ample clinical change that characterizes the initial hours of care. Few patients (17, 3.7%) were classified after 24 h as having a SpO_2_/FiO_2_ < 190 and PEEP < 10 cm H_2_O (group III), in accordance with previous observations in which only few patients with a worsening oxygenation are managed with low levels of PEEP [4]. The use of a different SpO_2_/FiO_2_ cutoff (150, corresponding with 100 mm Hg as used in the Berlin definition for ARDS to classify severe ARDS) did not essentially change the results. This underlines the rationale of the 24 h re-classification.

The comparisons between risk groups emphasize the improvement in risk stratification after 24 h but also suggest that the proposed classification by SpO_2_/FiO_2_ and PEEP in four risk groups is dependent on other factors. The fact that differences between group III and the other groups did not reach significance could be explained in part by the small number of patients in this group after 24 h. Also interestingly, differences in outcomes between groups separated by PEEP were not significant (group I vs. II and group III vs. IV). This does not weaken the improvement in risk stratification after 24 h, but probably suggests that our findings are driven more by SpO_2_/FiO_2_ than by PEEP. Hence, although PEEP is an attractive stratification tool due to its ubiquity, alternative variables might result in a better classification when combined to the SpO_2_/FiO_2_ ratio.

The high mortality rates, especially in the lower risk groups, are high, but confirm those from previous investigations [4, 5]. Re-classification after 24 h lead to lower mortality rates in the lowest risk group, but still the proportion of patients that died was high. This too is in line with findings from the earlier studies [4, 5]. Noticeable differences were seen in APACHE IV, arterial pH, plasma lactate levels in the first 24 h, and vasopressor use between the risk groups, and all could, at least in part be associated with the significant differences in mortality rates. In fact, the post hoc multivariate analysis showed that stratification based on SpO_2_/FiO_2_ and PEEP looses it predictive capability when controlled for APACHE IV scores, plasma lactate levels, arterial pH, blood pressure levels and vasopressor therapy. This at least suggests that persistence of hypoxemia after 24 h could be a surrogate, e.g., overall disease severity but also underlines the weakness of a simplified classification system. Nevertheless, we think that the proposed risk stratification may still be useful in settings where only pulse oximetry is available for monitoring and where laboratory examinations to compute disease severity scores are lacking.

The results of this study suggest that the SpO_2_/FiO_2_ can accurately surrogate the PaO_2_/FiO_2_ in stratifying for mortality of adult patients with established ARDS in resource-rich settings. This simple approach may in particular be useful in settings where repeated blood gas analyses are difficult to obtain or unavailable, such as in pediatric patients [23] and resource-limited settings [42]. However, it must be acknowledged that oximetry may never fully replace arterial blood gas analyses completely. Indeed, acid–base status and PaCO_2_ levels are clinically important. However, we stress that pulse oximetry is consistently present in low-resource settings while blood gas analyzers are scarce, if not completely absent. Moreover, several point-of-care devices allow to measure capillary or venous acid–base, potentially alleviating the need for an arterial blood sample and expensive blood gas analysis devices.

The SpO_2_/FiO_2_ may also turn useful in identifying patients at risk for ARDS. It has already been shown to be a useful independent indicator of ARDS in the Kigali criteria for ARDS, in which SpO_2_/FiO_2_ and lung ultrasound were used instead of PaO_2_/FiO_2_ and chest radiography [42]. Further studies in resource-rich and resource-poor settings are still needed to assess the utility of these adapted criteria in patients at risk for ARDS.

Limitations of our analysis include its single-center design and the lack of standardization of the SpO_2_ measurements, due to their retrospective collection. Indeed, standardization of ventilatory settings at baseline and after 24 h, and thus calculation of the PaO_2_/FiO_2_, may be crucial for appropriate stratification and patient selection bias minimization [43], and the same may apply for SpO_2_-based markers. While we tried to minimize potential errors by increasing the number of SpO_2_ values collected, we are aware that potential artifactual SpO_2_ values or ones resulting from acute events unrelated to the disease process may have been included (such as patient–ventilator asynchrony, obstruction of endotracheal tubes, suctioning and hemodynamic instability). We also did not collect information on skin pigmentation, motion artifacts, skin perfusion, ambient light, methemoglobinemia and carboxyhemoglobinemia levels—factors that all may be associated with the accuracy of SpO_2_ measurements. Its retrospective design allowed us neither to determine the type of pulse oximeter used nor to capture the positions of the probes used in individual patients. The minor but not quantifiable amount of measurements from a different probe or location other than the conventional finger oximeter represents a methodological weakness, but we do not believe affects the outcome or correlation analysis in an important way. We focused on only two time points, i.e., the time of initial ARDS diagnosis and after 24 h. It may be possible that SpO_2_/FiO_2_ and PEEP at other time points add to risk stratification. Finally, re-classification after 24 h means that patients who died in the first 24 h are missed. The same happens with the small proportion of patients who are extubated or transferred before 24 h. We also excluded patients with a PaO_2_/FiO_2_ > 200 mm Hg; however, we do not believe this weakens our results. We did this for the two following reasons. Patients classified as having mild ARDS [1] are very ‘heterogeneous’ with respect to outcomes. Moreover, the milder degree of oxygenation impairments in these patients increases the number of patients with SpO_2_ values > 97%, above which the relationship between PaO_2_ and SpO_2_ becomes weak.

## Conclusions

SpO_2_/FiO_2_ and PaO_2_/FiO_2_ have a strong linear relationship. In contrast to risk stratification at initial ARDS diagnosis, risk stratification using SpO_2_/FiO_2_ and PEEP at 24 h leads to risk groups with worsening outcomes. Despite the fact that the association between risk group assignment and outcome is confounded by several factors, risk stratification using SpO_2_/FiO_2_ and PEEP could be useful and practical, especially in settings where repeated blood analyses are difficult or impossible.

## Additional file

**Additional file 1.** SF and PEEP in ARDS_Electronic Supplementary Material.

## Abbreviations

SpO_2_: peripheral capillary oxygen saturation
FiO_2_: fraction of inspired oxygen
PaO_2_: arterial oxygen partial pressure
PEEP: positive end-expiratory pressure
ARDS: acute respiratory distress syndrome
ICU: intensive care unit
VFD: ventilator-free days
APACHE: acute physiology and chronic health evaluation
LIPS: Lung Injury Prediction Score
ROC: receiver operating characteristic
AUC: area under the curve
AECC: American–European Consensus Conference on ARDS

## References

[1] Ranieri VM, Rubenfeld GD, The ARDS Definition Task Force* (2012). Acute respiratory distress syndrome: the Berlin Definition. JAMA.

[2] Hernu R, Wallet F, Thiollière F (2013). An attempt to validate the modification of the American-European consensus definition of acute lung injury/acute respiratory distress syndrome by the Berlin definition in a university hospital. Intensive Care Med.

[3] Villar J, Pérez-Méndez L, López J (2007). An early PEEP/FIO trial identifies different degrees of lung injury in patients with acute respiratory distress syndrome. Am J Respir Crit Care Med.

[4] Villar J, Fernandez RL, Ambros A (2015). A clinical classification of the acute respiratory distress syndrome for predicting outcome and guiding medical therapy*. Crit Care Med.

[5] Bos LD, Cremer OL, Ong DSY (2015). External validation confirms the legitimacy of a new clinical classification of ARDS for predicting outcome. Intensive Care Med.

[6] Rice TW, Wheeler AP, Bernard GR (2007). Comparison of the SpO/FIO ratio and the PaO/FIO ratio in patients with acute lung injury or ARDS. Chest.

[7] Chen W, Janz DR, Shaver CM (2015). Clinical characteristics and outcomes are similar in ARDS diagnosed by oxygen saturation/FiO_2_ ratio compared with PaO_2_/FiO_2_ ratio. Chest J.

[8] Parvathaneni K, Belani S, Leung D (2017). Evaluating the performance of the pediatric acute lung injury consensus conference definition of acute respiratory distress syndrome. Pediatr Crit Care Med.

[9] Klouwenberg PMCK, Ong DSY, Bos LDJ (2013). Interobserver agreement of centers for disease control and prevention criteria for classifying infections in critically Ill patients*. Crit Care Med.

[10] Bernard GR, Artigas A, Brigham KL (1994). Report of the American-European consensus conference on ARDS: definitions, mechanisms, relevant outcomes and clinical trial coordination. Intensive Care Med.

[11] Zimmerman JE, Kramer AA, McNair DS, Malila FM (2006). Acute Physiology and Chronic Health Evaluation (APACHE) IV: hospital mortality assessment for today’s critically ill patients. Crit Care Med.

[12] Gajic O, Dabbagh O, Park PK (2010). Early identification of patients at risk of acute lung injury: evaluation of lung injury prediction score in a multicenter cohort study. Am J Respir Crit Care Med.

[13] Quan H, Li B, Couris CM (2011). Updating and validating the Charlson comorbidity index and score for risk adjustment in hospital discharge abstracts using data from 6 countries. Am J Epidemiol.

[14] Khemani RG, Patel NR, Bart RD, Newth CJL (2009). Comparison of the pulse oximetric saturation/fraction of inspired oxygen ratio and the PaO_2_/fraction of inspired oxygen ratio in children. Chest.

[15] Pandharipande PP, Shintani AK, Hagerman HE (2009). Derivation and validation of SpO_2_/FiO_2_ ratio to impute for PaO_2_/FiO_2_ ratio in the respiratory component of the Sequential Organ Failure Assessment score. Crit Care Med.

[16] Ellis RK (1989). Determination of PO from saturation. J Appl Physiol.

[17] Brown SM, Grissom CK, Moss M (2016). Non-linear imputation of PaO_2_/FiO_2_ from SpO_2_/FiO_2_ among patients with acute respiratory distress syndrome. Chest.

[18] Khemani RG, Thomas NJ, Venkatachalam V (2012). Comparison of SpO_2_ to PaO_2_ based markers of lung disease severity for children with acute lung injury. Crit Care Med.

[19] Bellani G, Gattinoni L, Van Haren F (2016). Epidemiology, patterns of care, and mortality for patients with acute respiratory distress syndrome in intensive care units in 50 countries. JAMA.

[20] Serpa Neto A, Schultz MJ, Festic E (2016). Ventilatory support of patients with sepsis or septic shock in resource-limited settings. Intensive Care Med.

[21] Festic E, Bansal V, Kor DJ, Gajic O (2015). SpO_2_/FiO_2_ ratio on hospital admission is an indicator of early acute respiratory distress syndrome development among patients at risk. J Intensive Care Med.

[22] Group TPALICC (2015). Pediatric acute respiratory distress syndrome: consensus recommendations from the pediatric acute lung injury consensus conference. Pediatr Crit Care Med.

[23] Khemani RG, Rubin S, Belani S (2015). Pulse oximetry vs. PaO_2_ metrics in mechanically ventilated children: Berlin definition of ARDS and mortality risk. Intensive Care Med.

[24] Ferguson ND, Fan E, Camporota L (2012). The Berlin definition of ARDS: an expanded rationale, justification, and supplementary material. Intensive Care Med.

[25] Villar J, Pérez-Méndez L, Kacmarek RM (1999). Current definitions of acute lung injury and the acute respiratory distress syndrome do not reflect their true severity and outcome. Intensive Care Med.

[26] Papazian L, Forel J-M, Gacouin A (2010). Neuromuscular blockers in early acute respiratory distress syndrome. N Engl J Med.

[27] Guérin C, Reignier J, Richard J-C (2013). Prone positioning in severe acute respiratory distress syndrome. N Engl J Med.

[28] Mayordomo-Colunga J, Pons M, López Y (2013). Predicting non-invasive ventilation failure in children from the SpO_2_/FiO_2_ (SF) ratio. Intensive Care Med.

[29] Tripathi RS, Blum JM, Rosenberg AL, Tremper KK (2010). Pulse oximetry saturation to fraction inspired oxygen ratio as a measure of hypoxia under general anesthesia and the influence of positive end-expiratory pressure. J Crit Care.

[30] Sanz F, Dean N, Dickerson J (2015). Accuracy of PaO_2_/FiO_2_ calculated from SpO_2_ for severity assessment in ED patients with pneumonia. Respirology.

[31] Allardet-Servent J, Forel J-M, Roch A (2009). FiO_2_ and acute respiratory distress syndrome definition during lung protective ventilation*. Crit Care Med.

[32] Feiner JR, Weiskopf RB (2017). Evaluating pulmonary function: an assessment of PaO_2_/FiO_2_. Crit Care Med.

[33] Villar J, Pérez-Méndez L, Blanco J (2013). A universal definition of ARDS: the PaO_2_/FiO_2_ ratio under a standard ventilatory setting-a prospective, multicenter validation study. Intensive Care Med.

[34] López-Fernández Y, Azagra AM, de la Oliva P (2012). Pediatric acute lung injury epidemiology and natural history study: incidence and outcome of the acute respiratory distress syndrome in children. Crit Care Med.

[35] Bone RC, Maunder R, Slotman G (1989). An early test of survival in patients with the adult respiratory distress syndrome. The PaO_2_/FiO_2_ ratio and its differential response to conventional therapy. Prostaglandin E1 Study Group. Chest.

[36] Cooke CR, Shah CV, Gallop R (2009). A simple clinical predictive index for objective estimates of mortality in acute lung injury. Crit Care Med.

[37] Villar J, Pérez-Méndez L, Basaldúa S (2011). A risk tertiles model for predicting mortality in patients with acute respiratory distress syndrome: age, plateau pressure, and PaO_2_/FiO_2_ at ARDS onset can predict mortality. Respir Care.

[38] Bersten AD, Edibam C, Hunt T, Moran J (2002). Incidence and mortality of acute lung injury and the acute respiratory distress syndrome in three Australian States. Am J Respir Crit Care Med.

[39] Luhr OR, Karlsson M, Thorsteinsson A (2000). The impact of respiratory variables on mortality in non-ARDS and ARDS patients requiring mechanical ventilation. Intensive Care Med.

[40] Knaus WA, Sun X, Hakim RB, Wagner DP (1994). Evaluation of definitions for adult respiratory distress syndrome. Am J Respir Crit Care Med.

[41] Sloane PJ, Gee MH, Gottlieb JE (1992). A multicenter registry of patients with acute respiratory distress syndrome. Physiology and outcome. Am Rev Respir Dis.

[42] Riviello ED, Kiviri W, Twagirumugabe T (2016). Hospital incidence and outcomes of the acute respiratory distress syndrome using the Kigali modification of the Berlin definition. Am J Respir Crit Care Med.

[43] Villar J, Blanco J, del Campo R (2015). Assessment of PaO_2_/FiO_2_ for stratification of patients with moderate and severe acute respiratory distress syndrome. BMJ Open.

