# Supplementary material for: Risk stratification using SpO2/FiO2 and PEEP at initial ARDS diagnosis and after 24 h in patients with moderate or severe ARDS
Source: Ann Intensive Care. 2017 Oct 25;7:108. doi: 10.1186/s13613-017-0327-9 (PMC5656507; doi:10.1186/s13613-017-0327-9)
Supplement: Supplementary file 1 — Additional file 1. SF and PEEP in ARDS_Electronic Supplementary Material. [file 13613_2017_327_MOESM1_ESM.docx]

Electronic Supplementary Material to:

**Risk Stratification Using SpO_2_/FiO_2_ and PEEP at Initial ARDS Diagnosis and After 24 Hours in Patients with Moderate or Severe ARDS**

Luigi Pisani,^1,8^* Jan Paul Roozeman,^1^* Fabienne D. Simonis,^1,2^*

Antonio Giangregorio,^1^ Sophia M. van der Hoeven,^1,2^ Laura R. Schouten,^1,4^ Janneke Horn,^1^ Ary Serpa Neto,^1,6^ Emir Festic,^7^ Arjen M. Dondorp,^1,8^ Salvatore Grasso,^3^ Lieuwe D. Bos,^1,2,5^ and Marcus J. Schultz^1,2,8^ for the MARS consortium^#^

Authors with an ‘*’ contributed equally to this study

^#^Molecular Diagnosis and Risk Stratification of Sepsis (MARS) study

**Academic Medical Center, Amsterdam, The Netherlands**

^1^Department of Intensive Care

^2^Laboratory of Experimental Intensive Care and Anesthesiology (L·E·I·C·A)

^4^Department of Pediatrics

^5^Department of Pulmonology

**University of Bari Aldo Moro, Bari, Italy**

^3^Anesthesia and Intensive Care Unit, Department of Emergency and Organ Transplantation

**Hospital Israelita Albert Einstein, São Paulo, Brazil**

^6^Department of Critical Care Medicine

**Mayo Clinic, Jacksonville, Florida, USA**

^7^Pulmonary and Critical Care Medicine

**Faculty of Tropical Medicine, Mahidol University, Bangkok, Thailand**

^8^Mahidol–Oxford Research Unit (MORU)

Number of tables: 5 tables

Number of figures: 0 figures

**Contacts:**

LP: [luigipisani@gmail.com](mailto:luigipisani@gmail.com); JPR: [j.p.roozeman@amc.uva.nl](mailto:j.p.roozeman@amc.uva.nl); FDS: [f.d.simonis@amc.uva.nl](mailto:f.d.simonis@amc.uva.nl); AG: [antonio.giangregorio@hotmail.it](mailto:antonio.giangregorio@hotmail.it); SMvdH: [s.m.vanderhoeven@amc.uva.nl](mailto:s.m.vanderhoeven@amc.uva.nl); LRS: [l.r.schouten@amc.uva.nl](mailto:l.r.schouten@amc.uva.nl); JH: [j.horn@amc.uva.nl](mailto:j.horn@amc.uva.nl); ASN: [aryserpa@terra.com.br](mailto:aryserpa@terra.com.br); EF: [Festic.Emir@mayo.edu](mailto:Festic.Emir@mayo.edu); AJD: [ajen@tropmedres.ac](mailto:ajen@tropmedres.ac); SG: [Salvatore.grasso@uniba.it](mailto:Salvatore.grasso@uniba.it); LDB: [l.d.bos@amc.uva.nl](mailto:l.d.bos@amc.uva.nl); MJS: [marcus.j.schultz@gmail.com](mailto:marcus.j.schultz@gmail.com)

**Correspondence:**

Luigi Pisani

Department of Intensive Care

Academic Medical Center

Meibergdreef 9, 1105 AZ Amsterdam, The Netherlands

Email: [luigipisani@gmail.com](mailto:f.d.simonis@amc.nl)

**Table E1.** Intergroup comparisons for secondary outcomes at ARDS diagnosis and after 24 hours, using a SpO_2_/FiO_2_ cut-off of 190 and PEEP of 10 cm H_2_O

| Covariate | At ARDS diagnosis | | After 24 hours | |
| --- | --- | --- | --- | --- |
|  | OR (95% CI) | *P*–value | OR (95% CI) | *P*–value |
| ICU mortality |  |  |  |  |
| Group II vs. I | 1.25 (0.40 – 3.94) | 0.960 | 1.73 (0.87 – 3.46) | 0.165 |
| Group III vs. I | 0.90 (0.35 – 2.36) | 0.993 | 2.97 (0.76 – 11.82) | 0.172 |
| Group IV vs. I | 1.77 (0.81 – 3.86) | 0.235 | 5.05 (2.41 – 10.59) | < 0.001 |
| Group III vs. II | 0.72 (0.23 – 2.25) | 0.883 | 1.72 (0.43 – 6.82) | 0.743 |
| Group IV vs. II | 1.42 (0.53 – 3.82) | 0.797 | 2.92 (1.38 – 6.17) | 0.002 |
| Group IV vs. III | 1.96 (0.92 – 4.18) | 0.103 | 1.70 (0.42 – 6.96) | 0.762 |
| 30–days Mortality |  |  |  |  |
| Group II vs. I | 1.02 (0.35 – 2.94) | 1.000 | 1.17 (0.63 – 2.18) | 0.917 |
| Group III vs. I | 0.83 (0.35 – 1.95) | 0.942 | 2.82 (0.77 – 10.32) | 0.166 |
| Group IV vs. I | 1.30 (0.64 – 2.65) | 0.770 | 2.67 (1.33 – 5.36) | 0.002 |
| Group III vs. II | 0.82 (0.28 – 2.34) | 0.959 | 2.42 (1.55 – 9.03) | 0.311 |
| Group IV vs. II | 1.28 (0.50 – 3.28) | 0.903 | 2.28 (1.09 – 4.78) | 0.021 |
| Group IV vs. III | 1.57 (0.78 – 3.16) | 0.338 | 0.95 (0.24 – 3.67) | 0.999 |
| 90–days mortality |  |  |  |  |
| Group II vs. I | 0.60 (0.23 – 1.60) | 0.540 | 1.03 (0.59 – 1.79) | 0.999 |
| Group III vs. I | 0.78 (0.37 – 1.66) | 0.830 | 1.83 (0.51 – 6.60) | 0.610 |
| Group IV vs. I | 0.81 (0.43 – 1.55) | 0.843 | 2.14 (1.09 – 4.19) | 0.019 |
| Group III vs. II | 1.30 (0.49 – 3.41) | 0.899 | 1.79 (0.49 – 6.59) | 0.655 |
| Group IV vs. II | 1.35 (0.56 – 3.28) | 0.814 | 2.09 (1.02 – 4.26) | 0.040 |
| Group IV vs. III | 1.04 (0.56 – 1.94) | 0.998 | 1.17 (0.30 – 4.53) | 0.991 |
| 1–year mortality |  |  |  |  |
| Group II vs. I | 0.46 (0.18 – 1.22) | 0.168 | 0.93 (0.54 – 1.60) | 0.984 |
| Group III vs. I | 0.57 (0.27 – 1.22) | 0.222 | 1.58 (0.43 – 5.77) | 0.793 |
| Group IV vs. I | 0.63 (0.33 – 1.21) | 0.267 | 2.10 (1.06 – 4.16) | 0.029 |
| Group III vs. II | 1.23 (0.48 – 3.15) | 0.940 | 1.71 (0.46 – 6.37) | 0.716 |
| Group IV vs. II | 1.36 (0.58 – 3.22) | 0.786 | 2.26 (1.10 – 4.68) | 0.020 |
| Group IV vs. III | 1.11 (0.60 – 2.05) | 0.973 | 1.32 (0.33 – 5.26) | 0.952 |

Abbreviations: ARDS, Acute Respiratory Distress Syndrome; OR, Odds ratio; CI, confidence interval

**Table E2.** Distribution and outcomes of each subset of patients with ARDS at initial diagnosis and after 24 hours using a SpO_2_/FiO_2_ cut-off of 150 (corresponding to PaO_2_/FiO_2_ of 100) and PEEP 10 cm H_2_O.

|  | Group I | Group II | Group III | Group IV |  |
| --- | --- | --- | --- | --- | --- |
| **Outcome** | SpO_2_/FiO_2_ ≥ 150 and PEEP < 10 | SpO_2_/FiO_2_ ≥ 150 and PEEP ≥ 10 | SpO_2_/FiO_2_ < 150 and PEEP < 10 | SpO_2_/FiO_2_ < 150 and PEEP ≥ 10 | *P*–value |
| **At onset of ARDS (N)** | 146 | 139 | 41 | 130 |  |
| ICU mortality (%) | 16.4 | 24.5 | 26.8 | 33.8 | <0.001 |
| In–hospital mortality (%) | 39.0 | 37.4 | 43.9 | 41.5 | 0.550 |
| 30–days mortality (%) | 24.7 | 30.9 | 29.3 | 33.8 | 0.120 |
| 90–days mortality (%) | 43.8 | 41.0 | 43.9 | 40.8 | 0.677 |
| 1–year mortality (%) | 53.4 | 47.5 | 53.7 | 49.2 | 0.629 |
| VFD–28 (days) (IQR) | 20 (8 – 25) | 20 (0 – 23) | 18 (0 – 23) | 14 (0 – 22) | 0.593 |
| **After 24hours (N)** | 227 | 198 | 3 | 28 |  |
| ICU mortality (%) | 17.2 | 28.8 | 0 | 60.7 | <0.001 |
| In–hospital mortality (%) | 36.6 | 39.9 | 0 | 67.9 | <0.001 |
| 30–days mortality (%) | 26.0 | 29.8 | 0 | 60.7 | 0.001 |
| 90–days mortality (%) | 39.6 | 41.4 | 0 | 71.4 | 0.012 |
| 1–year mortality (%) | 48.5 | 49.0 | 33 | 78.6 | 0.018 |
| VFD–28(days) (IQR) | 22 (14 – 26) | 15 (0 – 22) | 6 (6 – 7) | 0 (0 – 13) | <0.001 |

Abbreviations: VFD–28, ventilator–free days and alive at day 28; IQR, interquartile range; CI, confidence interval**Table E3.** Inter-group comparisons for all subset of patients with ARDS, classified at initial diagnosis and after 24 hours using a SpO_2_/FiO_2_ cut-off of 150 (corresponding to PaO_2_/FiO_2_ of 100) and PEEP 10 cm H_2_O.

| Comparison | At ARDS diagnosis | | After 24 hours | |
| --- | --- | --- | --- | --- |
|  | OR (95% CI) | *P*–value | OR (95% CI) | *P*–value |
| ICU mortality |  |  |  |  |
| Group II vs. I | 1.65 (0.77 – 3.53) | 0.332 | 1.95 (1.09 – 3.47) | 0.017 |
| Group III vs. I | 1.86 (0.64 – 5.42) | 0.435 | – | – |
| Group IV vs. I | 2.60 (1.24 – 5.46) | 0.005 | 7.39 (2.63 – 31.07) | <0.001 |
| Group III vs. II | 1.13 (0.40 – 3.18) | 0.990 | – | – |
| Group IV vs. II | 1.58 (0.79 – 3.16) | 0.322 | 3.82 (1.38 – 10.59) | 0.005 |
| Group IV vs. III | 1.40 (0.50 – 3.86) | 0.833 | – | – |
| Hospital Mortality |  |  |  |  |
| Group II vs. I | 0.93 (0.50 – 1.74) | 0.992 | 1.15 (0.71 – 1.88) | 0.870 |
| Group III vs. I | 1.22 (0.49 – 3.04) | 0.942 | – | – |
| Group IV vs. I | 1.11 (0.59 – 2.08) | 0.974 | 3.63 (1.29 – 10.39) | 0.008 |
| Group III vs. II | 1.31 (0.52 – 3.28) | 0.874 | – | – |
| Group IV vs. II | 1.19 (0.63 – 2.25) | 0.898 | 3.18 (1.11 – 9.07) | 0.025 |
| Group IV vs. III | 0.91 (0.36 – 2.29) | 0.993 | – | – |
| 30–days mortality |  |  |  |  |
| Group II vs. I | 1.37 (0.69 – 2.70) | 0.632 | 1.21 (0.71 – 2.05) | 0.782 |
| Group III vs. I | 1.26 (0.46 – 3.45) | 0.931 | – | – |
| Group IV vs. I | 1.56 (0.79 – 3.09) | 0.330 | 4.40 (1.59 – 12.14) | 0.001 |
| Group III vs. II | 0.92 (0.34 – 2.50) | 0.997 | – | – |
| Group IV vs. II | 1.14 (0.59 – 2.22) | 0.956 | 3.64 (1.31 – 10.08) | 0.007 |
| Group IV vs. III | 1.24 (0.46 – 3.35) | 0.947 | – | – |

**Table E3 continued**

| Comparison | At ARDS diagnosis | | After 24 hours | |
| --- | --- | --- | --- | --- |
|  | OR (95% CI) | *P*–value | OR (95% CI) | *P*–value |
| 90–days mortality |  |  |  |  |
| Group II vs. I | 0.90 (0.48 – 1.64) | 0.962 | 1.08 (0.66 – 1.74) | 0.978 |
| Group III vs. I | 1.00 (0.40 – 2.49) | 1.000 | – | – |
| Group IV vs. I | 0.88 (0.47 – 1.65) | 0.954 | 3.80 (1.30 – 11.11) | 0.008 |
| Group III vs. II | 1.13 (0.45 – 2.82) | 0.987 | – | – |
| Group IV vs. II | 0.99 (0.53 – 1.87) | 1.000 | 3.53 (1.20 – 10.40) | 0.015 |
| Group IV vs. III | 0.88 (0.35 – 2.22) | 0.984 | – | – |
| 1–year mortality |  |  |  |  |
| Group II vs. I | 0.79 (0.43 – 1.44) | 0.742 | 1.02 (0.63 – 1.65) | 0.999 |
| Group III vs. I | 1.01 (0.41 – 2.50) | 1.000 | 0.53 (0.03 – 11.25) | 0.948 |
| Group IV vs. I | 0.85 (0.46 – 1.57) | 0.896 | 3.90 (1.19 – 12.79) | 0.018 |
| Group III vs. II | 1.28 (0.52 – 3.18) | 0.897 | 0.52 (0.02 – 11.05) | 0.944 |
| Group IV vs. II | 1.07 (0.57 – 2.00) | 0.992 | 3.78 (1.16 – 12.61) | 0.022 |
| Group IV vs. III | 0.84 (0.33 – 2.10) | 0.959 | 7.32 (0.29 – 187.73) | 0.379 |

Abbreviations: ARDS, Acute Respiratory Distress Syndrome; OR, Odds ratio; CI, confidence interval

**Table E4.** Multivariable model including all groups classified after 24 hours from ARDS diagnosis.

| Covariate | OR (95% CI) | *P*–value |
| --- | --- | --- |
| Arterial pH | 0.01 (0.00 – 0.22) | 0.003 |
| APACHE IV | 1.02 (1.01 – 1.02) | <0.001 |
| Plasma Lactate Level | 1.12 (1.05 – 1.19) | <0.001 |
| Arterial Blood Pressure* | 0.99 (0.97 – 1.01) | 0.396 |
| Use of Vasopressor | 0.57 (0.30 – 1.11) | 0.094 |
| Group I | 1 (Reference) | – |
| Group II | 0.55 (0.33 – 0.93) | 0.028 |
| Group III | 2.00 (0.53 – 7.72) | 0.301 |
| Group IV | 0.97 (0.49 – 1.87) | 0.915 |

Abbreviations: APACHE, Acute Physiology and Chronic Evaluation Score; OR, Odds ratio; CI, confidence interval;

*Minimum levels of systolic arterial blood pressure over the first 24 hours.

**Table E5.** Multivariable model considering only group IV versus group I after 24 hours from ARDS diagnosis.

| Covariate | OR (95% CI) | P–value |
| --- | --- | --- |
| Arterial pH | 0.02 (0.00 – 0.74) | 0.037 |
| APACHE IV | 1.02 (1.01 – 1.03) | 0.001 |
| Plasma Lactate Level | 1.09 (1.02 – 1.18) | 0.024 |
| Arterial Blood Pressure* | 0.98 (0.95 – 1.01) | 0.173 |
| Use of Vasopressor | 0.36 (0.16 – 0.82) | 0.015 |
| Group IV | 1.11 (0.55 – 2.20) | 0.775 |

Abbreviations: APACHE, Acute Physiology and Chronic Evaluation Score; OR, Odds Ratio; CI, confidence interval;

*Minimum levels of systolic arterial blood pressure over the first 24 hours
